# Development of an Anatomy-Mimicking, Wave Transport-Preserving Mock Circulation Loop for Evaluating Pulsatile Hemodynamics as Supported by Cardiovascular Assist Devices

**DOI:** 10.1007/s13239-025-00785-y

**Published:** 2025-04-22

**Authors:** Pong-Jeu Lu, Ming-Yao Chan, Steven Tsui, Tzung-Tza Shen, Jui-Chih Chang

**Affiliations:** 1 3R Life Sciences, Ltd., Kaohsiung, Taiwan; 2 Heart Science and Medical Devices Research Center, National Cheng Kung University, Tainan, Taiwan; 3 Department of Cardiothoracic Surgery, Royal Papworth Hospital NHS Foundation Trust, Cambridge, UK; 4 Department of Surgery, Hualien Tzu Chi Hospital, Buddhist Tzu Chi Medical Foundation, Hualien, Taiwan; 5 Department of Surgery, School of Medicine, Tzu Chi University, Hualien, Taiwan

**Keywords:** Mechanical circulatory support, Mock circulation loop, Pulsatile flow physiology, In-vitro hemodynamic testing, Circulatory assist device design

## Abstract

**Objective:**

Assessing circulatory hemodynamics in-vitro is crucial for cardiovascular device design before in-vivo testing. Current mock circulation loops (MCLs) rely on simplified, lumped-parameter hydraulic representations of human circulation. There is a need for a more sophisticated MCL that can accurately represent the human circulatory physiology and allow for critical assessment of device-supported hemodynamics.

**Methods:**

An anatomy-mimicking MCL design guided by one-dimensional flow models has been developed, using tree-like arterial casts to create a complex system. The MCL comprises cardiac simulators, systemic circulatory subsystems consisting of 46 connected arterial casts, and lumped venous and pulmonary components. A parameter tuning process was also developed to ensure that the simulated MCL baselines are consistent with targeted healthy or heart failure scenarios.

**Results:**

Blood pressure and flow waveforms in the thoracic aorta, upper and lower limb arteries and abdominal organs (kidney, liver, spleen, etc.) were reproduced and validated against published data. Complex afferent and efferent flows in cerebral circulation and phasic coronary flow subjected to myocardial compression effect were replicated with precision. Pulse wave behavior was authentically generated and compared favorably to the published in-vivo and in-silico results.

**Conclusion:**

This wave transport-preserving MCL is able to simulate pulsatile human circulatory hemodynamics with sufficient detail and accuracy. Complex cardiovascular device-intervened hemodynamics in large arteries and end organs can be systematically assessed using this new MCL, potentially contributing to a rapid and accurate in-vitro simulation to help advance device design and functional optimization.

## Introduction

Mechanical circulatory support (MCS) devices have been widely used in treating acute and chronic advanced heart failure that is refractory to medical therapy. In the device design and verification phases, mock circulation loop (MCL) testing [1] and animal trials have been instrumental tools. Typically, after initial in-vitro design verification and promising results achieved on system performance and reliability tests, expensive animal trials are conducted as the next step. However, with growing concerns for animal welfare [2], there is a renewed focus on adhering to the 3R principles of experimental animal use (reduction, refinement, and replacement) and minimizing their use as a design verification/validation tool.

The development of a MCL that more closely mimics human anatomy and provides high-fidelity human circulatory physiology is essential for further advancing the cardiovascular device design and improvement. Various MCL designs have been developed historically to simulate the hemodynamic conditions encountered in devices designed for treating advanced cardiac diseases. Most of these circulatory assist devices are directly attached to the heart, making the connected vascular system greatly simplified into lumped element (windkessel) models. Such lumped element MCLs were sufficient in assessing the design objectives regarding transvalvular flow characteristics, cardiac output production and ventricular preload/afterload pressure conditions [3–8]. In some advanced hybrid MCLs [9–11], autoregulation mechanism including Frank-Starling mechanism, cerebral and coronary autoregulatory responses [12], and/or baroreceptor influence on systemic vascular resistance (SVR) regulation [13] were incorporated to further improve the authenticity of the device-intervened hemodynamic characteristics.

The study of partial-support MCS [14–18] called for the addition of vascular modules in the proximal arterial segments [19, 20] to simulate devices deployed intra- or extra-vascularly. Moreover, along with the increasing trend of extra-corporeal membrane oxygenation (ECMO) applications, the need of adding peripheral arterial and venous vessel replicas to allow cannula insertion drove the MCL design to a more realistic representation of the vasculature [21].

The aim of the present work was to design and construct an MCL that has a more elaborate cardiac and vascular representation to assist device design and analyze the circulatory physiology influenced by the connected devices. Unlike classical MCL designs based primarily on the lumped-parameter modeling, the present MCL was constructed using one-dimensional (1-D) flow model [22–25] to guide loop design. Correct wave mechanics prevailing in large arteries was emphasized to improve in-vitro test fidelity. Design aspects including modular component design, vascular tree structure construction, hemodynamic performance validation, system operation and tuning method will be presented. Finally, demonstrations of circulatory pulse wave characteristics in large arteries and major organs will be shown to demonstrate the capability and accuracy of this newly constructed wave transport-preserving circulatory simulator.

## Materials and Methods

### Mock Loop Design Principle

For the present MCL design, an approach integrating 1-D flow model [22–25] with lumped-parameter model was adopted. Multiple elastic tube casts were assembled into tree-like structures to represent the pulsatile systemic circulation. Flow transport and wave propagation in large arterial system, hence, can be accurately represented using such tree-like elastic tube structures appended with terminal lumped resistances and compliances that reflect the truncated downstream vasculature effect [23, 25]. In this design, practical simplifications were made to focus on larger arteries relevant to device design or organ perfusion. The systemic circulation was modeled using silicone rubber casts with similar anatomic lumen geometries and compliance properties. For the pulmonary circulation, lumped modules of resistors and compliance chambers [26, 27] were used due to the primary focus on arterial hemodynamic interactions with mechanical circulatory assist devices.

**Fig. 1:**
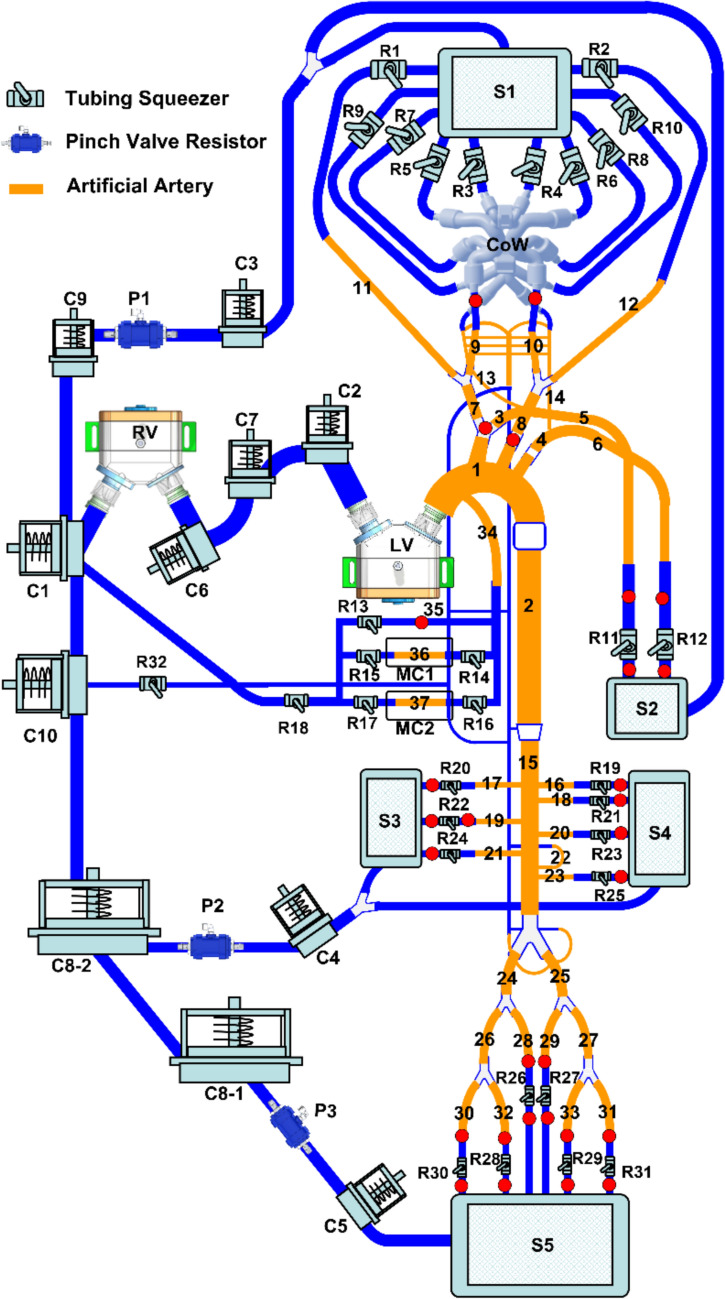
Schematic of the anatomic-mimicking mock circulation loop. Names and station/segment numbers of constituent components are provided in Table. 1**.** Silicone artificial arteries (orange) and smaller Tygon tubing arteries (blue) form the tree-like arterial system. Lumped pulmonary and venous components are interconnected with the arterial system by larger Tygon tubing (blue). LV, left ventricle; RV, right ventricle; CoW, Circle of Willis; P1–P3, pinch valve resistors; R1–R31, terminal resistors; C1–C10, compliance chambers; S1–S5, settling chambers; MC1, MC2, myocardial compression chambers; Red dots, pressure measurement sites

**Fig. 2:**
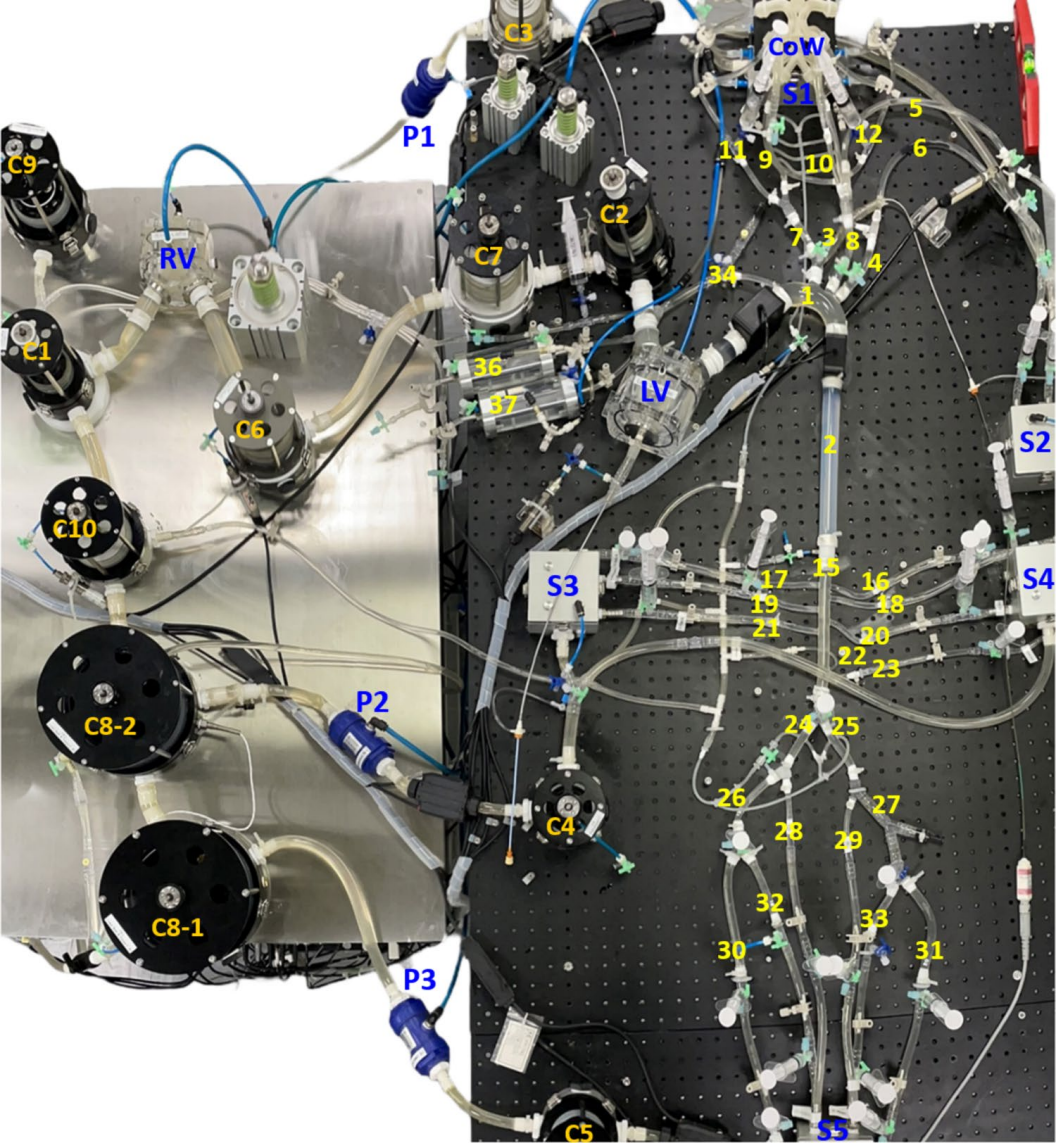
Picture of the anatomy-mimicking mock circulation loop. The numbers marked on the parts are defined in Table 1

The present MCL, shown in Figs. 1 and 2, consists of modular subsystems including cardiac simulators that simulate the functions of left ventricle (LV) and right ventricle (RV), as well as a tree-like systemic network comprising the thoracic aorta and the connected cerebral circulation, coronary circulation, spinal cord vasculature and aorto-iliac branches that supply blood to the various abdominal end organs and lower limb. The working blood-mimicking fluid (density: 1090 ± 50 kg/m^3^ and viscosity: 3.0 ± 0.5 mPa s) was prepared using a mixture ratio of 3:7 of glycerol to normal saline solution. The design highlights of each constituent subsystem are described in the following.

### Left/Right Cardiac Simulators

**Fig. 3:**
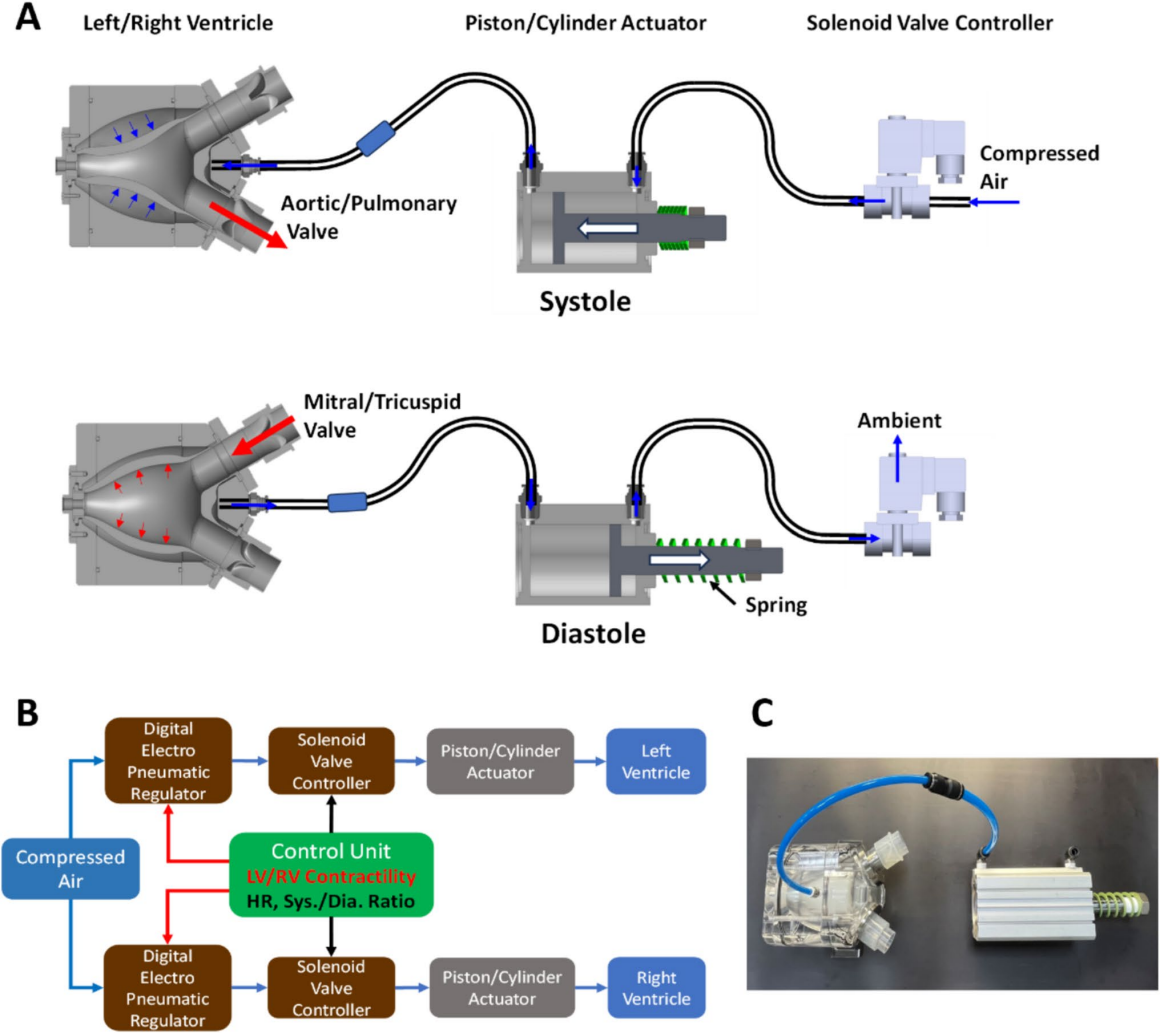
Cardiac simulator design. Ventricular contraction and relaxation are realized using a computer-controlled pneumatic driving system. A flexible blood sac is housed in a rigid-walled acrylic chamber in communication with a piston/cylinder actuator driven by a pneumatic regulator and air supply system. The inlet and outlet ports of the sac space (ventricle) are installed with two silicon tri-leaflet valves, respectively. **A** illustrated the schematic of the cardiac simulator design and the pneumatic ventricular actuator motion at systole and diastole. **B** depicts the control block diagram of pneumatic supply/regulation, driving flow and the commands sent from the control unit to actuate the ventricles. **C** shows the picture of the mechanical parts of the cardiac simulator

The LV and RV were actuated using a computer-controlled pneumatic system, as shown in Fig. 3. Contraction and relaxation of each ventricle was represented by an actively-driven elastic sac housed in a rigid chamber. The artificial sac was made of silicone rubber and has a heart-like appearance and volume that mimic a healthy or a diseased (hypertrophic or dilated) phenotype. The artificial sac was housed inside a transparent acrylic chamber in air communication with a piston/cylinder actuator driven by a pneumatic controller (Fig. 3A). Driving air was regulated by a solenoidal valve that controls the compressed air into the piston/cylinder actuator, resulting in a stroke piston motion to compress the sac in the chamber. The ventricular contraction was achieved by controlling the driving air pressure level, duration and actuation timing, in a beat-to-beat manner, via a computer-controlled pressure regulator in connection to the air supply system (Fig. 3B). During diastole, the solenoidal valve was switched to vent the cylinder to the ambient and the sac was passively filled while assisted by a tailored recoiling spring force (Fig. 3A). Appropriate spring constant can be selected to best match the desired diastolic pressure-volume relationship, simulating various ventricular filling characteristics (normal, dilated or stiffened myocardium).

The uni-directional intraventricular flows were accomplished using artificial tri-leaflet valves installed in the inflow and outflow tracts of the artificial ventricles. These artificial valves were made by mold injected silicone rubber. Unlike the conventional mock loop designs that either lack valves or use mechanical valves, these soft silicone valves can better reproduce valve closure-induced wave characteristics (Dicrotic notch) without generating spurious high-frequency pressure and flow oscillations commonly associated with mechanical valves or hydraulic pump.

### Tree-like Systemic Circulation Subsystem

**Fig. 4:**
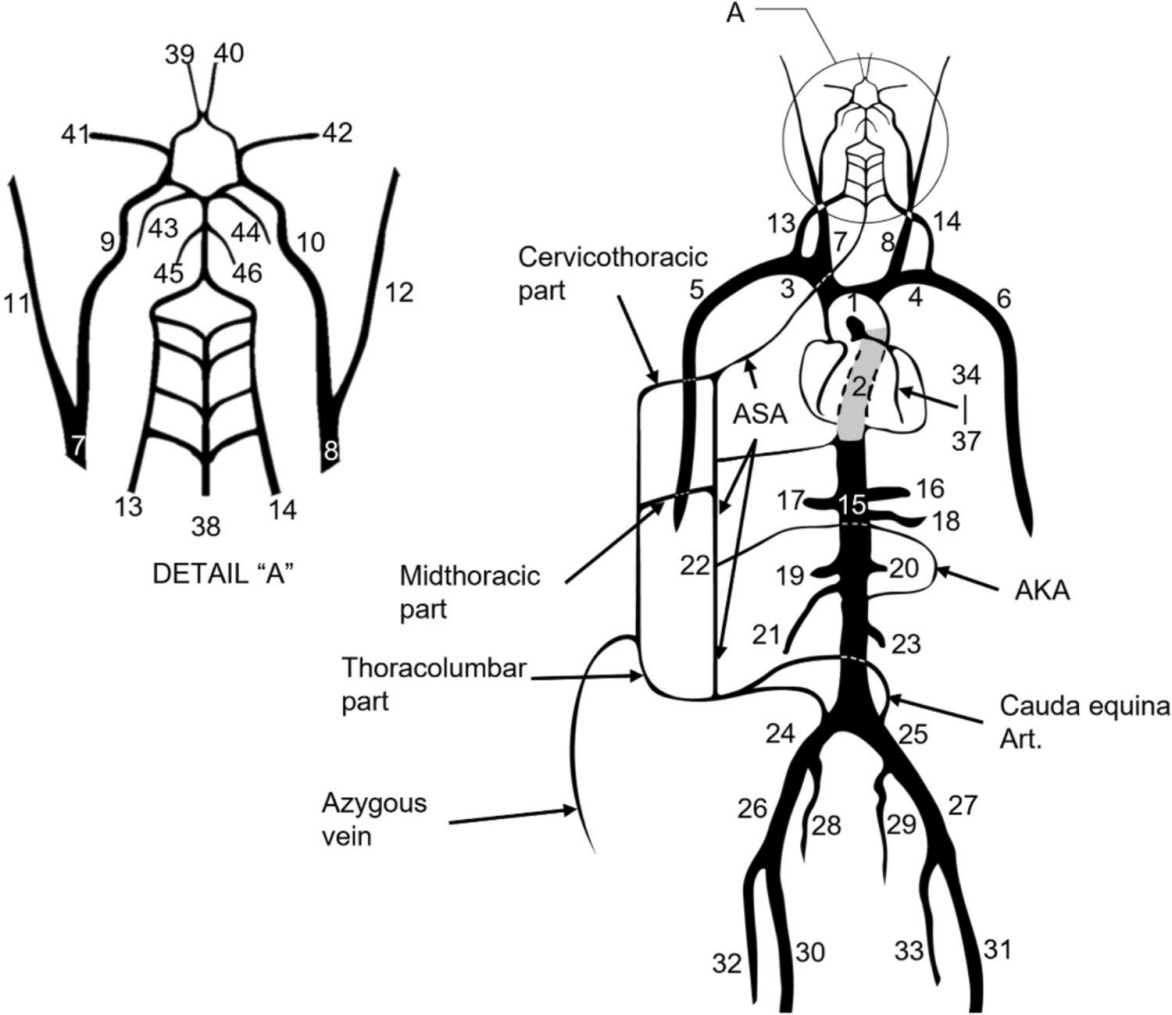
Schematic representation of the arterial tree replicas used on the present mock circulation loop. The segment numbers, names, and geometrical parameters are documented in Table 1. Spinal and intercostal circulations are simplified into lumped tubing parts as illustrated in the schematic. The lumped spinal/intercostal circulation parts are connected with the systemic arterial and venous subsystems to provide the afferent and efferent flows in the Circle of Willis as well as in the spinal circulation. AKA, artery of Adamkiewicz; ASA, anterior spinal artery

Figure 4 shows the arterial tree structure constructed for the present MCL, which includes 46 arterial casts (specifications given in Table 1) that were simplified from the adult human vascular anatomy. The arterial segments were made separately and assembled into tree-like structure using T- or Y-shaped connectors. The arterial segments are casting molded by silicone rubber and the distensibility of each vascular cast was approximated using the tube law [19, 24], in which the wall rigidity *Eh/r*^*0*^ (*E* is the Young’s modulus, *h* the wall thickness, and *r*^*0*^ the stress-free radius) was selected to make the analytically calculated tube compliance agree with the published target values. The physiological parameters used to design each arterial segment are given in Table 1. Our MCL model adopted healthy arterial geometry and compliance in the construction of the peripheral arterial tree.

**Table 1:** Physiological data of artificial arteries and terminal parameters used in MCL design

Nos.	Arterial segment	$l$ (cm)	$d_{0}$ (mm)	$d_{1}$ (mm)	$h$ (mm)	Compensated syringe air volume (ml)	Terminal resistance/compliance	
							$R_{T}$#/value (mmHg s/ml)	$C_{T}$ (10^-3^ ml/mmHg)
1	Aortic Arch	17.5	29	27	1.2			
2	Des. Aorta	15.0	22.2	22.2	1.5			
3	R. Subclavian A.	4.5	7.5	7.5	0.6	1.1		
4	L. Subclavian A.	4.5	7.5	7.5	0.6	1.1		
5	R. Brachial A.	34.0	6.5	6.5	0.5	38.0	**R11**/24.99	9.7
6	L. Brachial A.	34.0	6.5	6.5	0.5	38.0	**R12**/24.99	9.7
7	R. Common Carotid A.	5.0	9.4	9.4	0.6	14.5		
8	L. Common Carotid A.	5.0	9.4	9.4	0.6	16.5		
9	R. Internal Carotid A.	6.5	6.5	6.5	0.4	1.5		
10	L. Internal Carotid A.	7.5	6.5	6.5	0.4	1.5		
11	R. External Carotid A.	6.5	6.5	6.5	0.4	1.5	**R1**/12.50	
12	L. External Carotid A.	7.5	6.5	6.5	0.4	1.5	**R2**/12.50	
13	R. Vertebral A.	7.0	6.5	6.5	0.4			
14	L. Vertebral A.	7.0	6.5	6.5	0.4			
15	Abdominal Aorta	18.9	20	17.5	1.2			
16	Gastric A.	7.1	6.5	6.5	0.4		**R19**/19.50	4.6
17	Hepatic A.	5.0	6.5	6.5	0.4		**R20**/30.59	6.8
18	Splenic A.	6.5	6.5	6.5	0.4		**R21**/45.60	10.7
19	Superior Mesenteric A.	5.9	6.5	6.5	0.4	0.5	**R22**/7.84	26.6
20	L. Renal A.	2.5	6.5	6.5	0.4		**R23**/9.43	21.9
21	R. Renal A.	2.5	6.5	6.5	0.4		**R24**/9.43	21.9
22	Intercostal A.	-	-	-	-		**R32**/28.30	
23	Inferior Mesenteric A.	2.0	6.5	6.5	0.4		**R25**/58.06	3.6
24	R. Common Iliac A.	5.8	9.4	9.4	0.6			
25	L. Common Iliac A.	5.8	9.4	9.4	0.6			
26	R. External Iliac A.	7.0	7.5	7.5	0.5	11.2		
27	L. External Iliac A.	7.0	7.5	7.5	0.5	11.2		
28	R. Inner Iliac A.	3.0	6.5	6.5	0.4	3.6	**R26**/71.37	3.1
29	L. Inner Iliac A.	3.0	6.5	6.5	0.4	3.6	**R27**/71.37	3.1
30	R. Femoral A.	14.0	9.4	9.4	0.6	7.9	**R30**/23.14	9.6
31	L. Femoral A.	14.0	9.4	9.4	0.6	7.9	**R31**/23.14	9.6
32	R. Deep Femoral A.	8.0	7.5	7.5	0.5		**R28**/42.92	5.2
33	L. Deep Femoral A.	8.0	7.5	7.5	0.5		**R29**/42.92	5.2
34	Main Coronary A.	8.0	7.5	7.5	0.5		**R18**/20.4	
35	Sub-epicardium A.	8.0	4.78	4.78	1.59		**R13**/46.2-	
36	Sub-endocardium A.	8.0	7.5	7.5	0.5		**R14**/30.6 **R15**/12.6	
37	Endocardium A.	8.0	7.5	7.5	0.5		**R16**/19.2- **R17**/24.0	
	Cerebral Circulation							
38	Anterior Spinal A.							
39	R. Anterior Cerebral A.		4.78	4.78	1.59		**R3**/90.6	
40	L. Anterior Cerebral A.						**R4**/90.6	
41	R. Middle Cerebral A.						**R5**/59.9	
42	L. Middle Cerebral A.						**R6**/59.9	
43	R. Posterior Cerebral A.						**R7**/117.9	
44	L. Posterior Cerebral A.						**R8**/117.9	
45	R. Shunting A.						**R9**/136.8	
46	L. Shunting A.						**R10**/136.8	

$l$, arterial segment length; $d_{0}$, proximal lumen diameter; $d_{1}$, distal lumen diameter; $h$, lumen wall thickness; $R_{T}$, terminal resistance; $C_{T}$, terminal compliance; Des., descending; R., right; L., left; A., artery; C., compliance

The proximal and distal lumen diameters of most arterial segments were selected equal except for the ascending and descending aortas that had curved or tapered lumen diameter distributions. Owing to the simplification of arterial wall geometry and the limitation of silicone material property, the compliance in the manufactured tubular casts may be imprecise, which may cause discrepancy in the pulse wave propagation speed. Corrective compensation was made by adding compensated air volume to the original terminal compliance, provided in sum by a syringe capacitor installed at the distal end of each arterial branch cast. The measured total volume compliance of the vascular tree amounts to 0.741 ml/mmHg, which agrees with the empirical value documented by Olufsen [24].

Cerebral circulation was represented by a Circle of Willis (CoW) model connected with 4 affluent and 8 effluent arterial segments [25], as illustrated in Fig. 4. The vasculature downstream of the effluent arteries was represented by lumped terminal resistances and an overall compliance. Each terminal resistance can be adjusted using a tube squeezer. The effluent cerebral flows were issued, through separate Tygon tubing into a settling chamber, and collected by an upper body compliance chamber. The detail physiological data of the CoW arterial branches are documented in Table 1 (No. 38-46).

**Fig. 5:**
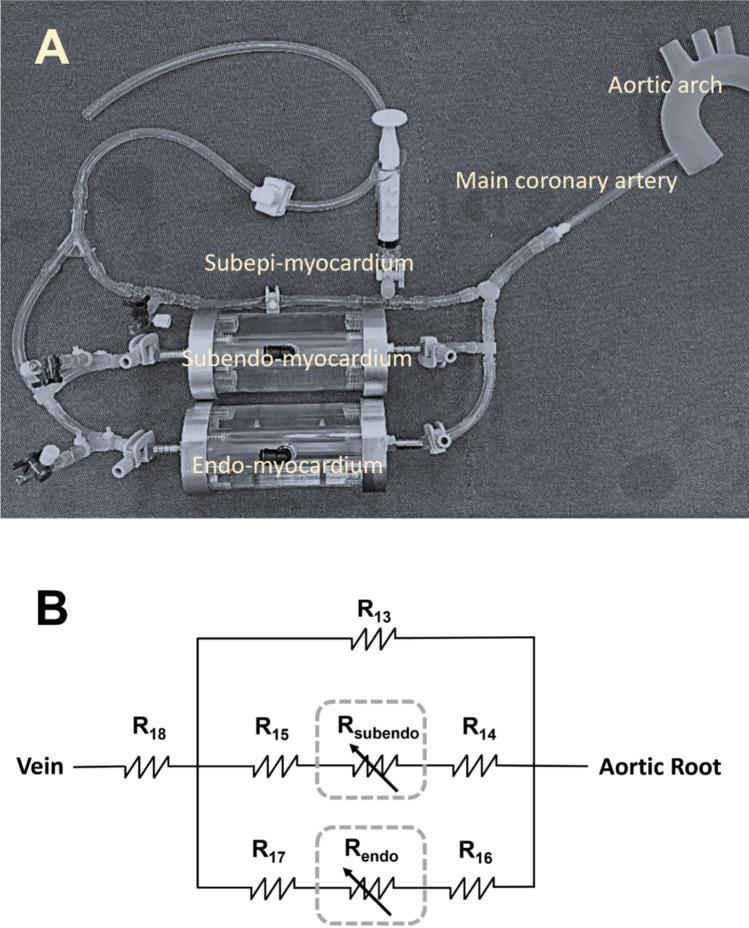
Triple-layered left coronary vasculature model (**A**) and the electric circuit analogue (**B**). Intramyocardial compression is imitated using collapsible silicon tubes housed in two air chambers (dash boxes), respectively. Driving pressures in the two air chambers are synchronized with the left ventricular driving system with regulated pressure drops to simulate the transmural muscular compression/relaxation effect. Resistances **R**_13_–**R**_18_ (see Table 1) are parameters used for the tuning of coronary flow distributions in the triple-layered myocardium model

Coronary circulation is subjected to myocardial forces, characterized by phasic differences in waveforms among arteries, arterioles, venules and veins [28, 29]. This phasic phenomenon comes primarily from the intramyocardial compression/relaxation effect exerted on numerous embedded vasculatures during ventricular systole/diastole. In order to simulate the phasic coronary flow phenomenon resulting from intramyocardial compression and relaxation, the present coronary circulation model employs a triple-layered lumped model, which divides the LV wall into layers of subepi-myocardium, subendo-myocardium and endo-myocardium. The vasculature in each myocardial layer was represented by a collapsible tube, whereas in each lumped layer the transmural compression force exerted on the embedded vascular wall varies. This proposed coronary model and its electric circuit analogue are shown in Fig. 5. The length, lumen geometry, wall thickness and resistances of the triple-layered coronary model are described in Table 1 (No. 34-37).

The intramyocardial compression effect was presently modeled using two air chambers, each being supplied with regulated time-varying air pressure corresponding to the subendo- or endo-myocardial transmural pressurization, respectively. A compression-free tube was employed to model the epi- and subepi-myocardial large arteries that were exposed to the atmosphere. Each compression chamber has a communication tube in connection to the LV pneumatic driver so that the intra-myocardial forces can be synchronized with the ventricular contraction and relaxation action. Linear transmural compression force distribution [28], varying linearly from ambient pressure to left ventricular pressure (LVP), was assumed and implemented using resistors to control the transmural pressure drops appearing in the modeled myocardial layers.

Fig. 4 also shows the abdominal aorta and the connected organs. This model simplifies the abdominal arteries into seven major lateral visceral branches and one intercostal branch [22, 23]. Three abdominal branches including hepatic, superior mesenteric and right renal arteries bifurcated from the abdominal aorta and merged into a right abdominal settling chamber. Similarly, four branches including gastric, splenic, left renal and inferior mesenteric arteries coalesced into the left settling chamber. Detail physiological data of the abdominal arterial segments and the terminal resistances and compliances are provided in Table 1 (No. 15-23).

The lower limb arterial branches, as shown in Figs. 1 and 4, begin as the continuation of the abdominal aorta and terminate in the left/right femoral and deep femoral arteries [22, 23]. A total of ten artificial tube segments were assembled into this limb division. Three pairs of lower limb branches including L/R external common iliac, L/R femoral and L/R deep femoral arteries merged into the lower limb settling chamber.

In the present MCL, blood supply to the spinal cord system was simplified into three interconnected lumped tubes, namely, the cervicothoracic, midthoracic and thoracolumbar parts as depicted in Fig. 4.

### Lumped Compliance Chambers

A compliance chamber was constructed to simulate the overall vascular compliance associated with a segmented circulatory domain. The present compliance chamber adopted the spring-based compliance design. The spring constant is inversely proportional to the compliance. The left and right atria in the present MCL were also represented by two passive compliance chambers. The locations of these compliance chambers are illustrated in Fig. 1, and the corresponding compliance values were determined based on Heldt et al. [26] and De Larrari et al. [27].

### Syringe Capacitor

A syringe with a specific amount of air volume was connected to the distal end of each artificial artery branch. The compliance provided by the air volume in the syringe is proportional to the air volume divided by the air pressure.

The terminal compliance represents the gross compliance associated with the truncated vasculature downstream of that artery. In some cases, the manufactured artificial tubular cast was stiffer than its represented native artery. To compensate for this modeling discrepancy, specific syringe capacitance was used. In the present MCL design, the air volume assigned to each syringe capacitor is the sum of the compensated volume compliance of the upstream artificial arterial tube plus the downstream terminal compliance.

### Terminal and Lumped Resistors

Terminal resistors were constructed using two Tygon tubing (1/4 or 3/16 inch ID, Saint-Gobain, Charny, France) segments, each internally packed with multi-luminal catheter inserts. This design allows for adjustable flow resistance in a linear fashion in the specified resistance ranges (50–150 and 5–80 mmHg/ml). By squeezing the tubing resistor using a squeezer, the cross-sectional area of the terminal resistor can be changed to provide the flow resistance desired.

The systemic tree-like arteries were categorized into upper body, abdominal and lower limb subdivisions. Three pneumatic pinch valves (IA 50647, Richway Industries, Janesville, IA) were used as the lumped resistors to control the flow ratios distributed among the upper body, lower limb and abdominal subdivisions in relation to cardiac output and, while also regulating the corresponding upstream vascular pressures. Pressure sensors (Sensormate Enterprise, Chang Hua, Taiwan) were mounted on the two sides of each pinch valve. The resistance of the lumped resistor, hence, can be determined (by dividing pressure differential by the flow passing the pinch valve) and adjusted according to the pre-calibrated flow resistance (Table 1). Pneumatic control valve (RP-1000 CKD, Aichi, Japan) was used to regulate the air pressure applied to the pinch valve, controlling flow resistance and flowrate past the pinch valve.

In each systemic circulatory subdivision, the tree-like 1-D flow parameters including vascular geometries, tube wall compliance and terminal compliance and resistances were modeled based on data in published literature [22–25]. Terminal resistances and compliances were determined in accordance with the average flowrate ratios assumptions, as suggested by Stergiopulos et al. [22] and Kolyva et al. [20].

Note that all the tree-like tubular arterial flows merged in-series to their receiving pinch valve resistors, respectively. When specifying the corrected terminal resistance specified at the distal end of each artery branch, the downstream pinch valve resistance must be subtracted from the referenced terminal resistance value shown in Table 1.

### Control Unit and Data Acquisition System

The MCL control unit includes an in-house developed user interface (UI), electronics and software system to drive the MCL and generate the required flow characteristics. The hardware of the control unit consists of three printed circuit board assemblies (PCBAs), including a MCL Controller PCBA, a Raspberry Pi3 (Raspberry Pi Ltd., Cambridge, England), and a Pneumatic Regulator Driving PCBA. When a user sets the simulation parameters, namely, heart rate, systolic ratio, and left/right ventricular contractility levels from the UI of the MCL system, the Raspberry Pi 3 embedded system will process, generate and send continuous and periodic command signals to the MCL Controller PCBA and the Pneumatic Regulator Driving PCBA to trigger the left/right pneumatic actuators according to the sent solenoidal valve opening/closing timings and a regulated pressure level, hence driving the loop circulation into motion.

The data acquisition (DAQ) system consists of a personal computer with two LCD displays, a data acquisition card (NI PCIe-6321 X Series DAQ, National Instrument, Austin, TX) and a BNC signal connector box (NI BNC-2090A, National Instrument, Austin, TX). This DAQ system can monitor up to 32 analog input channels with a maximum multi-channel sampling rate of 250 kS/sec and 16 bits resolution. This DAQ system can gather and process the measured flow and pressure signals. Pressure was measured using pressure transducer (Sensormate Enterprise, Chang Hua, Taiwan) with a measurement range and accuracy of − 50 to + 300 mmHg (± 5 mmHg). Flowrate was measured by transit-time flow probes (Transonic Systems, Ithaca, NY) with different collar sizes to allow the probes to be mounted on various vascular tubes. The software systems supporting the DAQ system include LabVIEW (National Instruments, Austin, TX) and NI Measurement & Automation Explore (MAX) (National Instruments, Austin, TX). The LabVIEW offers a programable graphical user interface that helps user visualize hardware configuration, acquire measured data and develop data analysis algorithms. The NI MAX, however, enables user to manage DAQ hardware, software and scaling of the calibrated sensors.

### Parameter Tuning Method and Process

There are systemic and pulmonary parameters involved in the tuning process when attempting to make the in-vitro MCL simulation relevant to certain human circulatory characteristics. Empirical parameter setting learned from lumped-parameter MCL tests can be taken as an initial starting point of tuning. All the terminal resistances and compliances shown in Table 1 were first selected based on a healthy adult data [22].

**Fig. 6:**
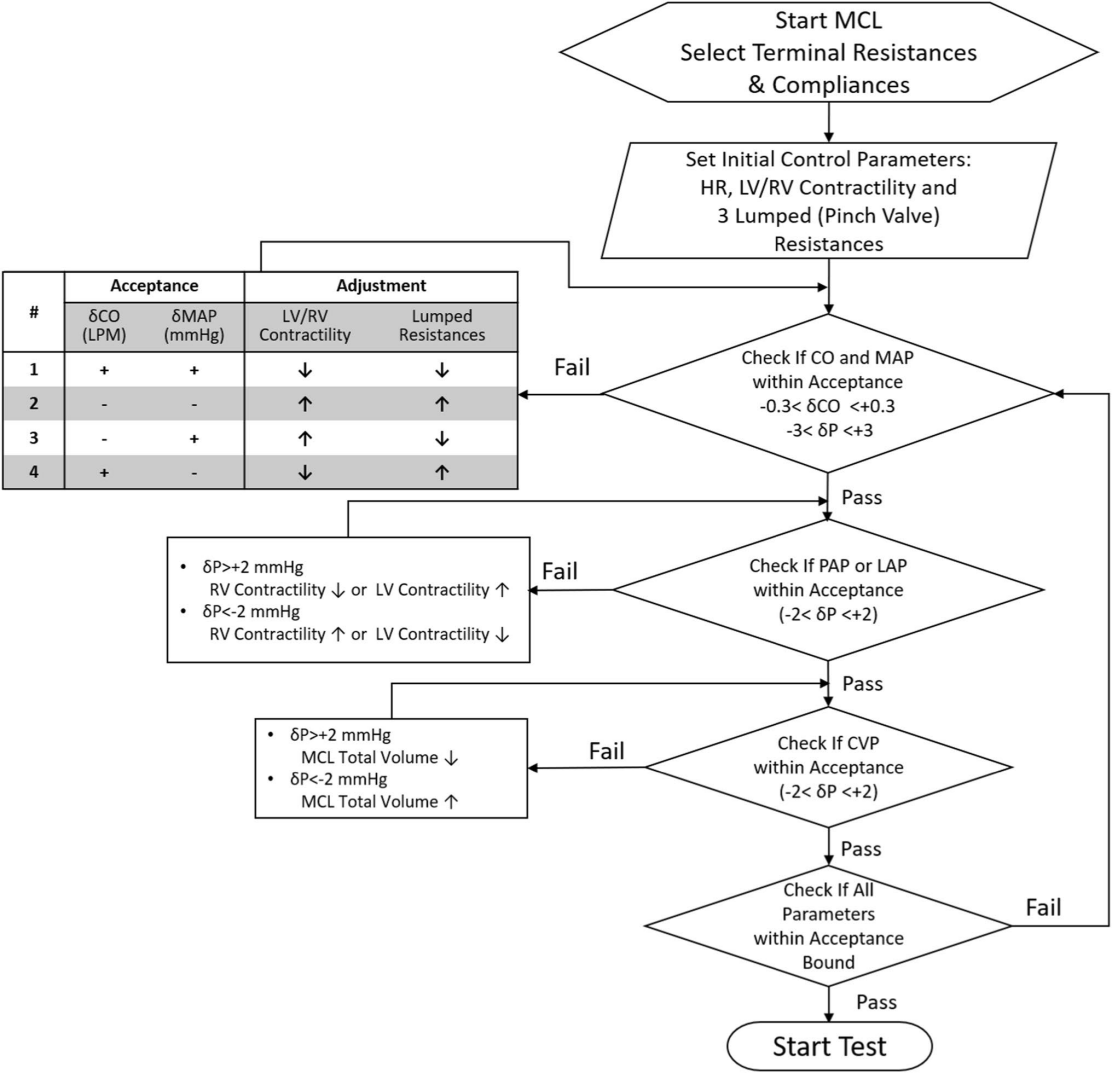
Flow chart showing the parameter tuning process of the present mock circulation loop

The parameter adjustment process involves multiple iterative tuning of the pinch valve resistances and LV/RV contractility levels while keeping the pre-selected terminal resistances and compliances literally unchanged. As shown in Fig. 6, the tuning process starts out from an initial setting with a given cardiac output (CO) and mean aortic pressure (MAP) as the target. From the database gathered in the present MCL library, the initial LV and RV contractility settings are selected in conjunction with a specified heart rate and systole/diastole ratio as input. Comparing the currently obtained CO and MAP to the corresponding targets, 4 possible ẟCO and ẟMAP combinations may occur. A rule-based adjustment in SVR and LV/RV contractility, as indicated in the first box in Fig. 6, will be facilitated to reduce ẟCO and ẟMAP within the preset error bound. The systemic vascular resistance (SVR) is computed as the current mean arterial and right atrial pressure differential (MAP-RAP) divided by CO*.* The tuning of SVR is carried out by changing the 3 pinch valve resistances. According to a predetermined ratios of flows flowing through the respective upper body, abdominal, and lower limb subdivisions, the pinch valve resistance change associated with each subdivision is assigned. Following the systemic parameter tuning, the pulmonary parameter tuning will be conducted to adjust the LV/RV contractility and the total circulation volume, according to the rules specified in the second and third boxes, to make the pulmonary arterial pressure (PAP), the left atrial pressure (LAP) and the central venous pressure (CVP) within each specified error bound. Such systemic and pulmonary tuning steps will be iteratively repeated until convergence was attained.

Since the tuning of compliance would not affect CO and MAP, the compliance adjustment can be performed as the last step. The total systemic compliance can be obtained using the current SVR and the measured diastolic decay rate on the aortic pressure waveform. The total terminal compliance can then be decided by subtracting the volume compliance of the entire arterial tree (0.741 ml/mmHg) from the currently obtained total systemic compliance. The updated change of the total terminal compliance can next be distributed to all the arterial terminals according to the aforementioned flow ratio rule [20, 22].

## Results

### Pulsatile Flow in Large Arteries

**Fig. 7:**
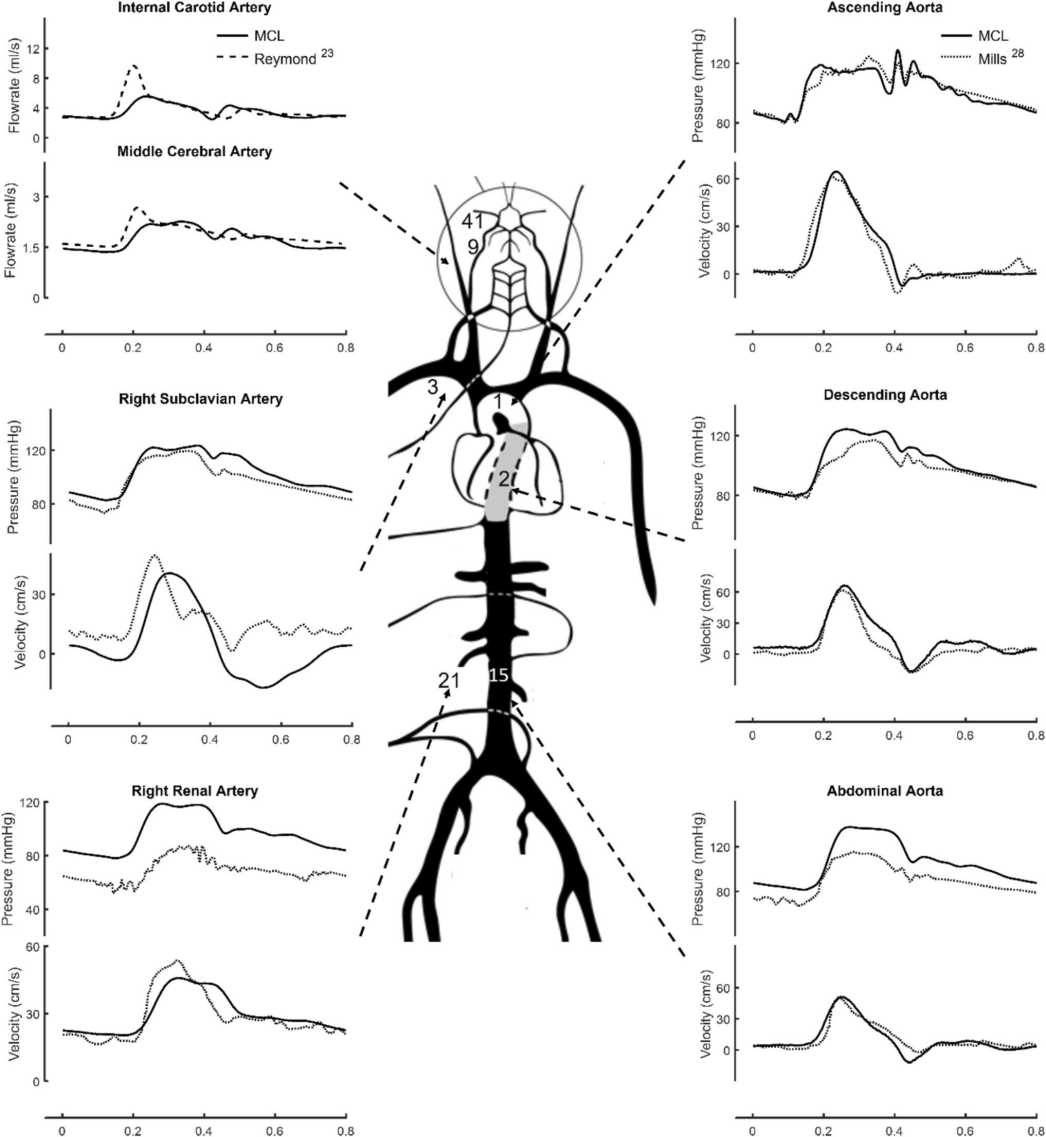
Comparison of the MCL-generated pressure and flowrate waveforms to those computational (Reymond et al. [23]) and clinically observed (Mills et al. [28]) results

The pulse wave phenomenon in large arteries has been successfully represented by the present MCL. The MCL-generated waveforms at different vascular segments of a patient with ischemic heart disease were compared against clinical [28] and computational [23] works, as shown in Fig. 7. The MCL parameter setting was first tuned to have a best fit of the MCL-generated ascending aortic pressure and flow waveforms to those of the simulated patient [28]. The hemodynamic waveforms at other vascular segments were generated accordingly on the MCL. During the tuning process, the terminal resistances and compliances assigned to the arterial branches were based on a healthy subject [22, 23].

In general, the characteristic wave mechanics in large arteries was well captured on the present MCL, which agreed reasonably well with the clinically observed waveforms [28]. Large discrepancy is seen in the right renal arterial pressure. However, Mills et al. [28] reported a sensor error was committed and the clinical right renal arterial data was replaced from a different patient. This indicates that the present MCL result in right renal artery might not be wrong. Of note, there are time lags of the peripheral pressure and flow waveforms relative to those appearing in the ascending aorta, reflecting a finite pulse wave speed involved in the pulse wave propagation in the present MCL.

High-frequency pressure oscillation was absent, indicating that spurious, non-physiological wave reflections were largely minimized. Furthermore, the renal and cerebral flows were characterized by non-negative values, showing the compliance effect of the elastic arteries. As shown by these MCL-generated central arterial waveforms, it is evident that antegrade flow was simulated during LV systole, which was further augmented by the recoil of the stretched vascular wall during diastole.

### Cerebral Circulation

Cerebral circulation, which was not represented in sufficient detail in conventional MCLs, was simulated in great details in the present MCL. In the upper left panel of Fig. 7 a comparison of flow waveforms in internal carotid and middle cerebral arteries were shown with qualitative consistency to the computational results [23]. Moreover, the flow perfusions in major arteries of CoW were compared in excellent agreement with the clinical measurements and the computational results [23, 25], as demonstrated in Table 2.

**Table 2.** Comparison of MCL cerebral flow with in-vivo and in-silico results

Nos.	Segment	Mean Flow Rate, *ml/min* (% to CO)			
		Reymond [23] In-vivo	Reymond [23] In-silico	Alastruey [25] In-silico	Present MCL
	CO	6180 (100%)	5760 (100%)	5710 (100%)	5300 (100%)
34	Main Coronary A.				159 (3.00%)
5/6	Arm			280.6 (4.87%)	240 (4.53 %)
7/8	CCA	390 (6.31%)	324 (5.63%)	N/A	315 (5.94%)
13/14	VA	72 (1.17%)	72 (1.25%)	N/A	65 (1.23%)
9/10	ICA	216 (3.5%)	234 (4.06%)	N/A	195 (3.68%)
11/12	ECA				120 (2.26%)
41/42	MCA	150 (2.43%)	114 (1.98%)	103 (1.80%)	100 (1.89%)
39/40	ACA	N/A	N/A	69.6 (1.21%)	66 (1.25%)
43/44	PCA	N/A	N/A	54.1 (0.95%)	51 (0.96%)
45/46	Shunting A.	N/A	N/A	N/A	43 (0.81%)

CO: cardiac output; CCA: common carotid artery; VA: vertebral artery; ICA: internal carotid artery; ECA: external carotid artery; MCA: middle cerebral artery; ACA: anterior cerebral artery; PCA: posterior cerebral artery. N/A: not available.

### Coronary Circulation and Wave Intensity Analysis

**Fig. 8:**
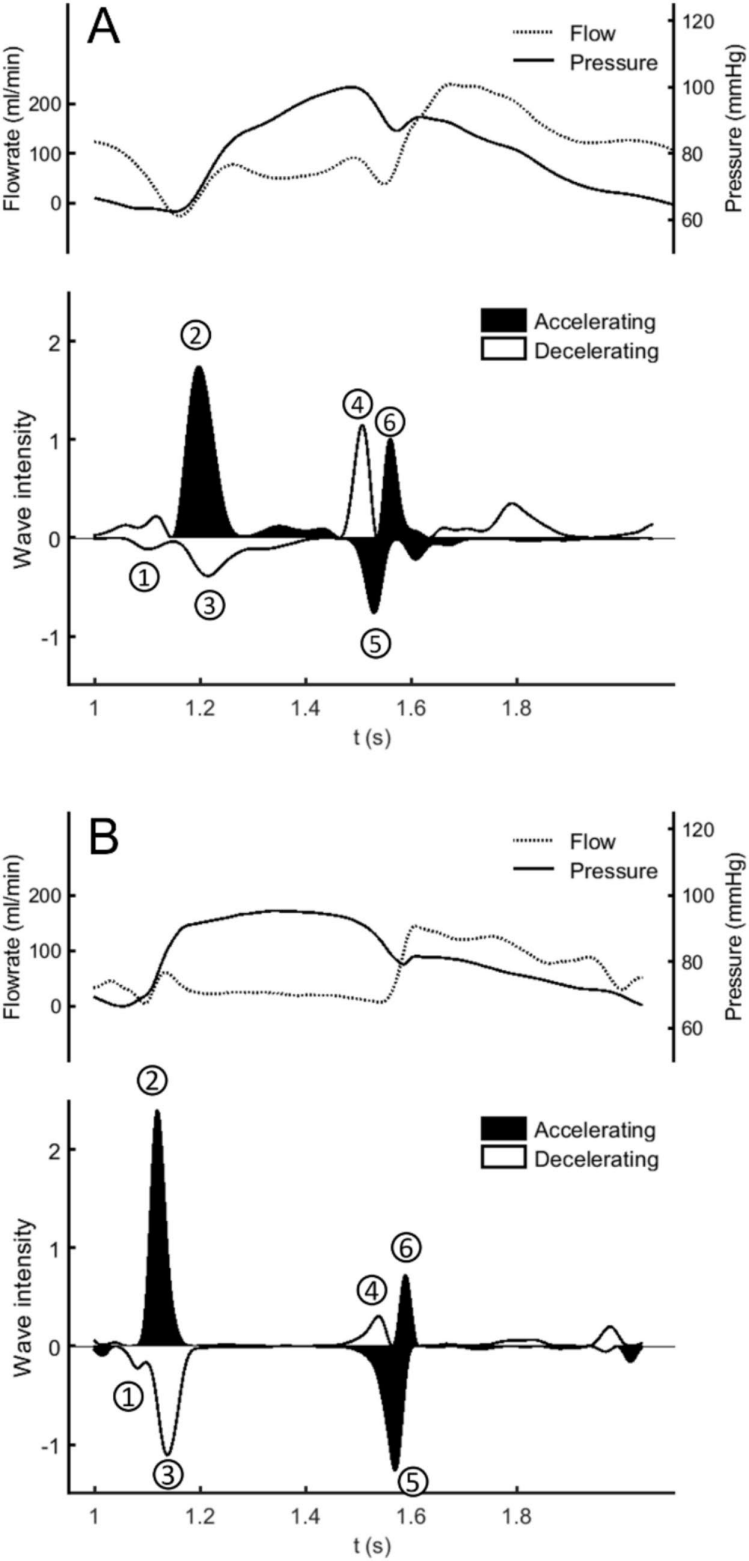
Phasic coronary flow and wave characteristics analyzed using Wave Intensity Analysis (WIA); MCL (**A**) vs. animal experiment (**B**) results. Characteristic coronary waves labeled with numbers can be found in Davies [30]. Wave 2 is the dominant forward-traveling pushing wave resulting from early ventricular contraction. Wave 5 represent the dominant backward-traveling “suction” wave, which is the major coronary perfusion mechanism attributed to vasculature sudden expansion during early diastolic phase

Phasic coronary circulation was authentically simulated in the present MCL (see Fig. 8). The phasic coronary flow [29, 30], with a nearly 180-degree phase shift to the systemic flow in aorta, was correctly represented. It was observed that coronary flow was mainly generated by the suction wave [30] created at the initial diastolic phase. Wave intensity analysis (WIA) [31–33] was employed to further delineate the phasic flow wave characteristics as compared to in-vivo data [34]. Notice that the WIA is strictly derived from the fundamental fluid conservation laws, which is adopted herein as the metrics to quantify the arterial pulse energy strength at a spatial point of interest. With the aid of WIA, pulse wave can be split into forward (+) and backward (-) propagating waves with their intensities given by

1 $$dI_{ \pm } = \pm \frac{1}{4\rho c}\left( {dP \pm \rho cdU} \right)^{2}$$

2 $$I_{ \pm } = \mathop \smallint \limits_{beat}^{{}} dI_{ \pm }$$

in which, $\rho$ is the fluid density, *c* is the wave speed, *P* is the pressure, *U* is the flow velocity and *d* represents the difference operator taken over a time interval of 0.004 s.

The six compression and decompression dominant waves [30, 34] characterizing the coronary circulatory phenomena were well reproduced (Fig. 8A). At the initial systolic phase, the LV contraction and aortic valve opening generates the first coronary perfusion peak (wave 2) together with a strong wave reflection (wave 3) due to the constricted coronary vasculature in the myocardium. Suction wave 5, the primary mechanism responsible for coronary perfusion, shows a forward accelerated, decompressed (suction) wave contributed by the rapid expansion of the coronary arteries due to myocardial relaxation around late systole and early diastole. The effect of aortic valve closure (Dicrotic notch) generates the forward accelerated compression wave (wave 6) that augments the coronary perfusion subsequent to the suction wave 5. To the knowledge of the authors, the present MCL is the first bench facility that can reproduce the coronary perfusion characteristics in compliance with the coronary phasic flow physiology.

### Heart Failure with Rotary Pump Support

The use of continuous-flow left ventricular assist devices (LVADs) or rotary pumps have been plagued with unresolved device-induced complications (stroke, gastrointestinal bleeding, aortic insufficiency, etc.) [35]. Diminished vascular pulsatility was thought to be the main causal factor of these complications [36]. Rotary pump speed modulation in an attempt to regain pulsatile physiology was adopted as a direction of improvement [37]. To this end, it requires a quantitative assessment to evaluate if an effective pulse improvement is achievable by a specifically designed speed modulation scheme. A MCL that can reproduce high-fidelity pulsatile physiology, hence, holds a key position for such advanced design objectives to be critically assessed.

**Fig. 9:**
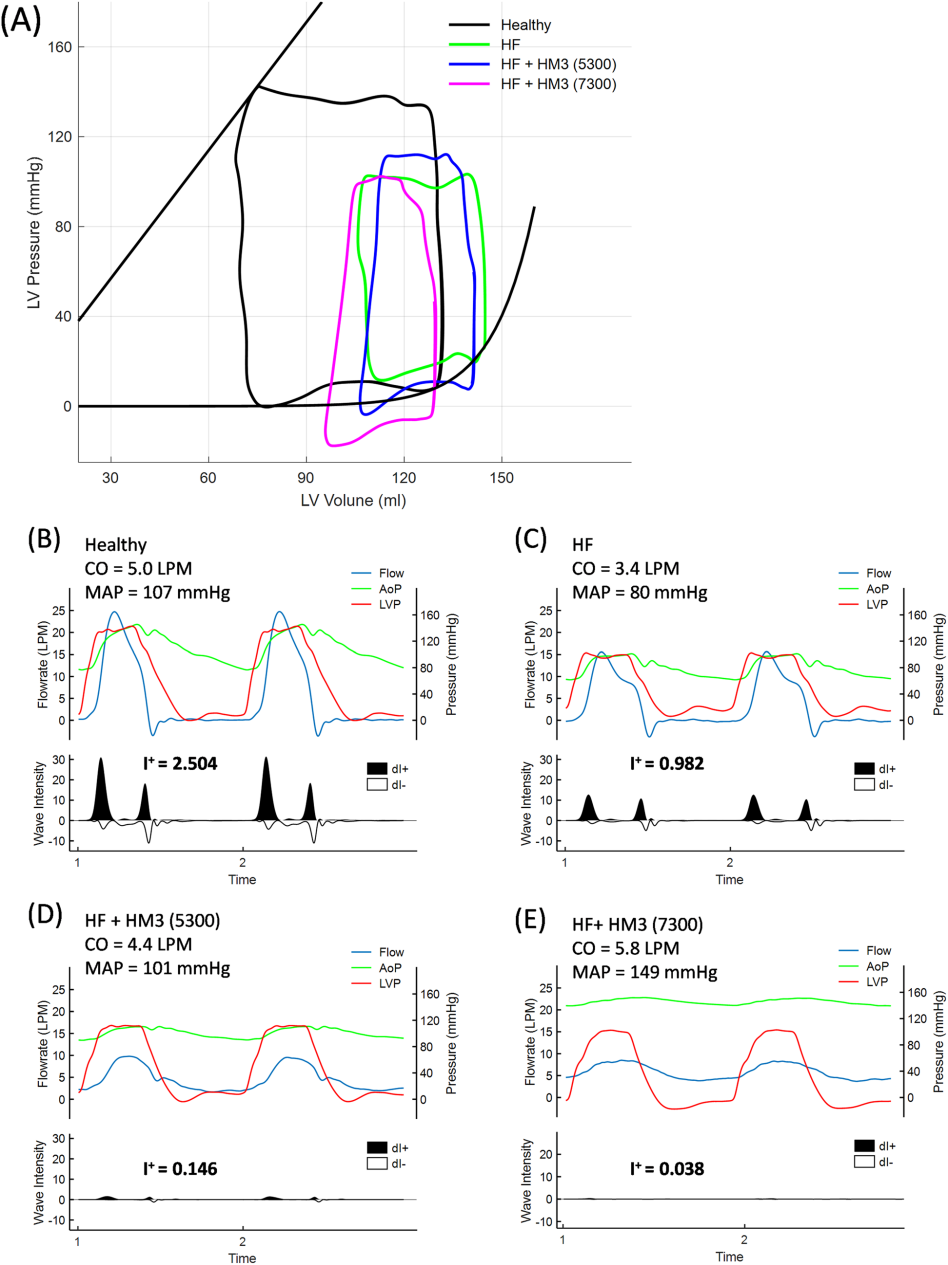
Hemodynamic simulation of healthy, heart failure, and heart failure supported by rotary pump left ventricular assist device (LVAD). **A** shows the left ventricular pressure-volume loops of a heart with and without LVAD support. **B**–**E** illustrate the pressures, flow and wave intensities corresponding to a healthy heart (**B**); a heart failure control (**C**); a heart failure supported by rotary pump with support level barely allowing aortic valve opening (**D**), and a heart failure having full rotary pump support with aortic valve closed

Fig. 9 illustrates a simulated case of heart failure (HF) (CO: 3.4 LPM, MAP: 80 mmHg) supported by a HeartMate 3 (HM3, Abbott Cardiovascular, North Plymouth, MN) rotary pump on the present MCL. The HeartMate 3 was connected from LV apex to ascending aorta, and HF was created by decreasing the LV/RV contractility and increasing the heart rate settings on the MCL. A Millar conductance catheter (Millar Inc., Houston, TX) was inserted via the mitral valve into the LV to evaluate the pressure-volume (PV) loop characteristics under healthy heart, heart failure control, and heart failure with speed-modulated pump supports. It is shown in Fig. 9A that, as HF is induced, PV loop shifts in the upper-right direction with a reduced loop area (LV stroke work). The HeartMate 3 support is able to decompress the distended LV, as evidenced from the left-downward shifted PV loops. Note that the HeartMate 3 supported PV loops were taken from the non-pulse periods to keep the presented PV loops clean. The corresponding LVP, AoP, and aortic flow (AoF) waveforms, with and without pump support, are shown in Fig. 9B–E, in which both forward- and backward-propagating WIs are illustrated to quantify the associated vascular pulsatility. It is observed that vascular pulsatility greatly diminishes when supported by HeartMate 3.

The total forward-propagating WI, *I*_*+*_, quantifies the LV-imparted pulse energy in a contractile beat. When comparing pump-supported cases to the unassisted HF control, *I*_*+*_ values were drastically reduced to 6 and 1.5% at the rotational speeds of 5300 and 7300 RPM, respectively.

In Fig. 9D illustrated an assisted condition (5300 RPM) which barely allows aortic valve opening. It is observed that the CO is slightly enhanced to 4.4 LPM from the unassisted HF baseline of 3.4 LPM. This case clearly showed that, for severe HF, rotary pump support effectiveness may be significantly compromised if aortic insufficiency is to be mitigated by a down-modulation of the rotational speed.

### Heart Failure Supported by IABP Counterpulsation

To date, IABP has been the most widely used modality in HF treatment. The balloon counterpulsation reduces LV contraction afterload while augmenting myocardial and organ perfusion. However, a thorough, quantitative in-vivo assessment of balloon counterpulsation efficacy is hard to perform. Traditional lumped-parameter based mock loops or numerical computation codes cannot provide reliable evaluation because such lumped models lack the ability to correctly simulate pulse wave propagation phenomena in arteries.

**Fig. 10:**
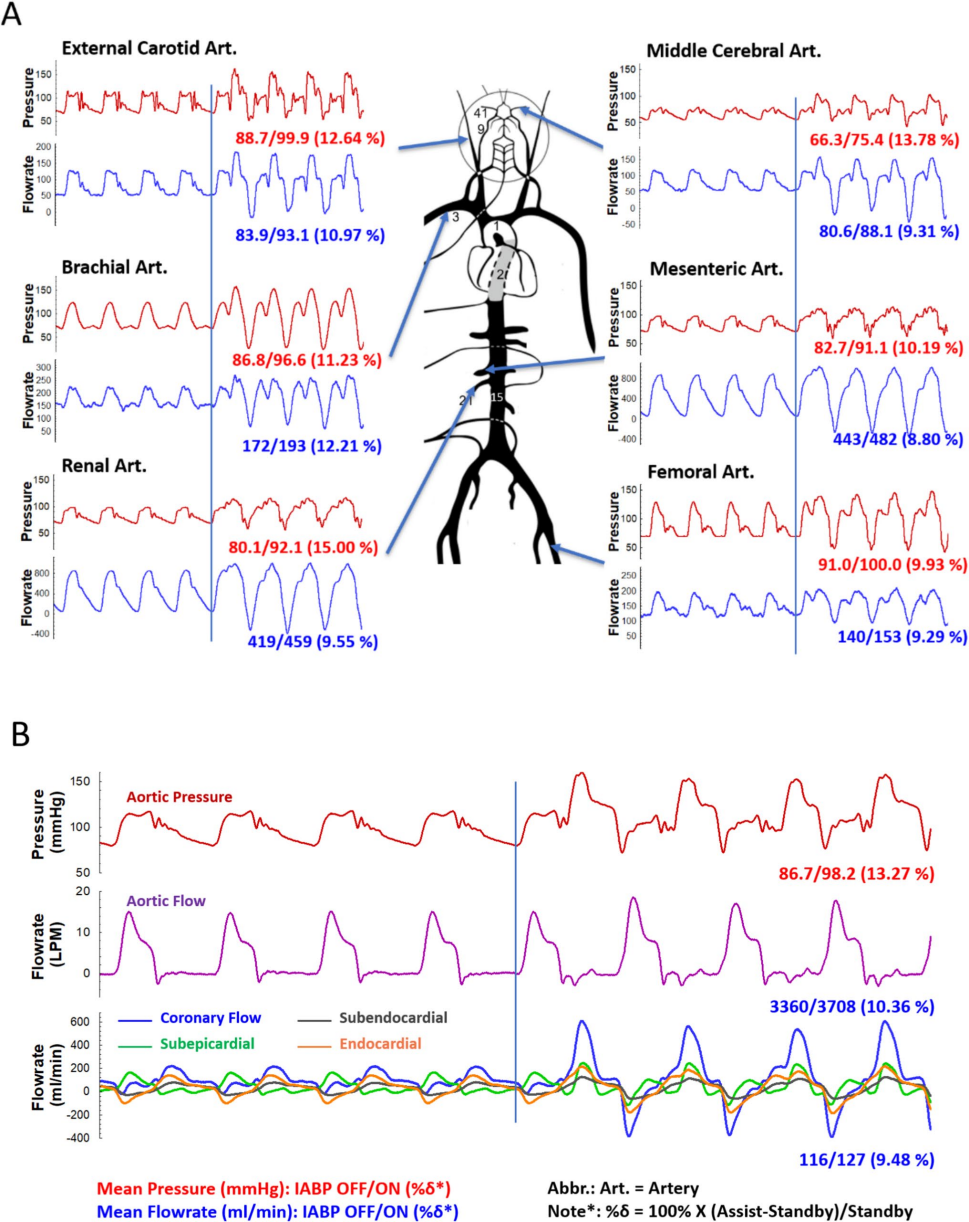
Simulation of heart failure (CO = 3.3 LPM, MAP = 90 mmHg, HR = 85 BPM) supported by intra-aortic balloon pump (IABP). **A** shows the pressure and flow waveforms at representative end organs and arteries under counterpulsatile IABP support (assist ratio 1:1, full augmentation). The time traces show IABP-standby and IABP-assisted waveforms separated by a line of start. The time-averaged pressure and flow enhancements are expressed as baseline/assisted (% increment). Units of flow and pressure are ml/min and mmHg, respectively. **B** illustrates the pressures and flows measured in the ascending aorta as well as in the coronary triple-layered (subepi-, subendo- and endo-myocardium layers) myocardial model. The time-varying resistance, resistor and compliance parameters are those adopted in Fig. 5.

We conducted an IABP-supported HF scenario aiming to stratify the support efficacy down to the organ level. The balloon catheter (40 cc Linear 7.5 Fr. IAB Catheter, Getinge, Sweden) was inserted from the artificial femoral artery and disposed in the descending aorta above the renal arterial branch. The driver console (Maquet Datascope CS300, Getinge, Sweden) delivered 1:1 assist ratio support with full augmentation. The results are illustrated in Fig. 10, which shows IABP standby and assisted circulation using pressure and flow waveforms.

Regarding the assisted circulation in vital end organs, the present MCL particularly provides a beat-to-beat waveform representation accompanied by integrated time-average values of mean pressure and flow (Fig. 10A). It is shown in the waveforms that IABP counterpulsation generates reversed flow during unloading (balloon deflation) and elevates flow during augmentation (balloon inflation). The net gains in flow perfusion in end organs remain positive, such as approximately 12–13% in brain, 10% in kidney, 9% in mesentery and lower limb. This waveform description of support characteristics reveals the sensitivity of balloon deflation timing in the assisted hemodynamics. Clinically, “blood steal” in brain was observed due to overly strong unloading. Although inflation/deflation timing optimization is not within the scope of the present study, the wave-preserving ability of the present MCL demonstrates that such advanced hemodynamic performance studies can be conducted on the benchtop facility.

In panel B we present the gross CO and AoP waveforms and further stratify myocardial perfusion into subepi-, subendo- and endo-myocardium layers. The clinically observed counterpulsatile pressure waveforms were reproduced with high-fidelity. Similar to what were observed clinically, the CO enhancement was moderate (10.36%) and both end-diastolic pressure and peak systolic pressure were lowered signifying the contraction unloading effectiveness.

As IABP is standby, the complex phasic coronary flow patterns in different myocardial layers are vividly illustrated in Fig. 10B. For example, during LV systole after aortic valve opening, the epi-myocardium received antegrade perfusion (positive flow) contributed by the initial systolic pressure rise while the endocardium experienced retrograde blood expulsion (negative flow) due to intramyocardial compression on the embedded vessels [29, 30]. In addition, the phasic myocardial perfusion attributed to the diastolic “suction” of the coronary flow in the early diastolic period is observed on the MCL. This suction-induced coronary flow results from the relaxation of the intramyocardial compression force and the sudden expansion of the coronary vascular lumen [30]. Moreover, it is known that, for patients suffering myocardial ischemia, the endocardium is often the most vulnerable region where the infarct lesion occurs. Perfusion enhancement measured at the epi-myocardial coronary artery is not equal to what the infarct ischemia is supplied, as shown by the amplitudes of the flow profiles in the subepi- and endo-myocardium layers, respectively.

It should be noted that the present coronary perfusion assist associated with balloon counterpulsation is just a qualitative demonstration of balloon-on and standby comparison. The loop parameters need to be validated against known clinical data to justify its final usability and clinical relevance.

## Discussions

The objective of the present work was to construct an in-vitro test facility, aimed at simulating human circulatory hemodynamics with high-fidelity. It is believed that, with the aid of this comprehensive, pulsatile physiology compatible MCL, advanced design objectives and device support optimization can be critically evaluated. Contrary to the lumped-parameter design guideline previously adopted in most MCLs, the present MCL development used 1-D flow model and anatomy-mimicking tree-like arterial casts to assist the loop design. As a result, an authentic reproduction of the pulsatile circulatory physiology in large arteries and end organs was achieved. Such MCL can be conveniently used as research or development platform to help predict or analyze the complex device-intervened hemodynamics that was difficult to evaluate via expensive animal models.

It is worth mentioning that the intricate flow inertia and spurious wave reflection problems [3] prevailing in traditional rigid-walled lumped-parameter MCLs was substantially eliminated in our MCL simulations. The present compliant, reflection-resistant vascular tree structure [24] and soft silicone cardiac valves effectively overcame these basic loop design difficulties. By mimicking the native vascular anatomy, a total of 46 arterial tree-like structure was formed. Around the vascular bifurcation junctures, the luminal diameter ratios of parent and daughter arteries and the branching angles naturally inhibits wave reflection as pulse waves pass down the tree-like structure [24]. This design eliminates the need for the troublesome inertance tuning process required in lumped-parameter MCLs to minimize spurious wave reflection.

As shown in Figs. 7 and 8, the MCL-generated pressure and flow waveforms closely mimic the pulse wave characteristics in human. Such genuinely reproduced pulsatile circulatory physiology will be instrumental in assisting the future pulsatile MCS design and control algorithm upgrade. In addition, the greater sophistication in arterial representation allows perfusions in individual end organs be assessed with quantitative accuracy. This may significantly reduce the need for in-vivo validation of device design.

Various cardiac conditions can be reconstructed using the present MCL. Both ventricular function and ventriculovascular coupling can be tuned to replicate the targeted HF scenario. The rotary pump-supported dilated cardiomyopathy shown in Fig. 9 is a representative case. Rotational speed modulation and the resultant flows regarding whether vascular pulsatility can be regained to the level of clinical significance was simulated and critically evaluated using WIA. Today, new pulsatile LVAD inventions with design focus on generating clinical pulsatile support are currently in development [38, 39]. The characteristics of device-produced pulsatility can be quantitatively assessed using the present MCL.

Temporary extracorporeal life support or percutaneously delivered axial-flow pumps are increasingly used as a result of the revised organ allocation priority [40]. It remains unclear how the percutaneously delivered flow, antegrade or retrograde, would adversely disturb or redistribute the original blood perfusion in organs and vasculature in a quantitative manner; or how the patient-specific support level should be properly administered in the course of treatment or weaning. Prior to clinical MCS intervention, in principle, all these prominent safety and performance related issues should be examined and quantified. This deeper understanding in device-intervened circulatory flow can potentially be gained and evaluated in advance using the present MCL.

The present MCL design is deeply rooted in human circulatory physiology and anatomy. The cardiac simulator and tree-like arterial network are direct inspirations from the human circulatory system, making it easier for clinicians to understand the links between hydraulic abstraction and clinical circulatory behaviors. This intuitive connection makes the MCL an ideal tool for a wide range of applications, including cardiovascular education and training. Medical students can use the MCL to gain hands-on experience with complex circulatory systems, while device designers can leverage its capabilities to optimize their designs and improve patient outcomes.

Furthermore, the present MCL can serve as a valuable research facility for practitioners involved in various MCS device applications. By simulating real-world scenarios and testing different hypotheses, researchers can gain deeper insights into the behavior of the human circulatory system and develop more effective device solutions for patients with cardiovascular disease.

The advancement of mechanical circulatory support systems has reached a stage requiring more sophisticated in-vitro test facility to better imitate the human circulatory anatomy and physiology. Inclusion of vascular tree-like structure and cardiac simulators that can closely mimic systole and diastole are essential.

The aim of reconstructing interested human circulatory physiology or pathophysiology for mechanical support devices design and optimization was initially achieved using the present loop design method. It is important to note that prior to any intended device evaluation the baseline hemodynamics needs to be validated. The example shown in Fig. 7 is just a demonstration that indicates that, by appropriate system parameters assignment, the target circulation characteristics can be replicated.

At present, cardiac and vascular parameter tuning was manually carried out following a tuning strategy outlined in Fig. 6. In the future MCL advancement this tuning process can be automated by installing computer-controlled hardware and software systems on the cardiac simulator and the lumped resistors and compliance chambers. Both feedback loop circuits and control logics associated with the parameter searching process illustrated in Fig. 6 will be developed to accomplish this autonomous loop tuning system.

Human circulation-related autoregulation has yet been incorporated in the present MCL. To further upgrade the loop emulation fidelity, cardiac and vascular autoregulation including, but not limited to, Frank-Starling law, baroreceptor control of vascular resistance, autoregulation of coronary and cerebral flow as well as respiration-influenced cardiorespiratory interaction can be added using the present loop configuration structure as a groundwork.

## Data Availability

The raw results of this study are available upon reasonable request from the corresponding author.
